# Chagas disease treatment efficacy markers: experiences from a Phase III study with nifurtimox in children

**DOI:** 10.3389/fpara.2023.1229467

**Published:** 2023-09-22

**Authors:** Ulrike Grossmann, Maria-Luisa Rodriguez

**Affiliations:** ^1^ Research and Development Pharmaceuticals, Bayer AG, Berlin, Germany; ^2^ Research and Development Pharmaceuticals, Bayer AG, Wuppertal, Germany

**Keywords:** Chagas disease, pediatric patients, nifurtimox, ELISA, IHA, qPCR

## Abstract

Determining the success of antitrypanosomal therapy for Chagas disease is challenging, particularly in the chronic phase of the disease, because seropositivity persists for a long time after successful antitrypanosomal treatment and is known to be related to the duration of *Trypanosoma cruzi* infection. Seroconversion to negative by two or more conventional serologic tests is the currently accepted measure of efficacy, and studies suggest no significant change in seropositivity if left untreated. However, there is no guidance for industry on how to establish the effectiveness of drugs intended for the treatment of Chagas disease. Due to the lack of validated sensitive, specific, easy-to-use markers that allow early monitoring of the efficacy of antitrypanosomal treatment in an efficient manner, we used seroreduction measured by two conventional enzyme-linked immunosorbent assays in addition to the currently accepted criterion for what constitutes a cure, seroconversion to negative, as a surrogate parameter for efficacy in a Phase III pediatric trial with nifurtimox. The measures for confirmation of the antitrypanosomal efficacy of nifurtimox were discussed with US FDA. In this perspective article, we present our experiences obtained from a pediatric study on Chagas disease with an established drug using a surrogate efficacy parameter in addition to the established criterion for a cure.

## 1 Introduction

Chagas disease (American trypanosomiasis) is a parasitic disease caused by the flagellate protozoan *Trypanosoma cruzi*. The disease has two clinical phases—an acute phase and a chronic phase—and the chronic phase is subdivided into indeterminate and determinate (symptomatic) forms. If left untreated, about two-thirds of infected people will remain in the chronic indeterminate stage, experiencing no symptoms or long-term sequela but carrying a hidden infection. However, in 20% to 30% of cases, serious progression occurs about 10 years to 30 years after the initial infection with *T. cruzi* (Perez-Molina and Molina, 2018), which can be life-threatening.

In this chronic symptomatic form of the disease, cardiac involvement is the most frequent and severe type of organ involvement. Chagas heart disease occurs in 10% to 30% of cases and is associated with a worse prognosis (Alvarez-Hernández et al., 2018; Meymandi et al., 2018). The exact mechanism of progression of Chagas disease to the determinate form is not fully understood; several factors including the persistence of high parasitemia and tissue parasitism have been discussed and may be a determining factor in the development of Chagas cardiomyopathy (Nunes et al., 2013). Gastrointestinal involvement in the chronic symptomatic form of Chagas disease is less common, and most often presents as megaesophagus and megacolon, characterized by hypertrophy of the muscle layers and progressive loss of contractile capacity (Alvarez-Hernández et al., 2018). No biomarkers are available to predict the risk of Chagas disease progression.

Chronically infected individuals in the indeterminate stage may transmit the *T. cruzi* infection either by the congenital route or by blood or organ donation and, therefore, initiation of antitrypanosomal treatment as soon as possible after diagnosis of acute or chronic Chagas disease is an important goal. Currently, only two compounds, nifurtimox and benznidazole, are available for the specific antiparasitic treatment of *T. cruzi* infection (Balouz et al., 2017). Elimination of the parasite through subsequent antitrypanosomal therapy is considered essential to prevent or delay severe complications and to prevent congenital transmission (Irish et al., 2022). It is recognized that an inflammatory immune response is triggered and sustained by *T. cruzi*, and it has been postulated that the parasite persistence and immunological mechanisms are inextricably related to myocardial aggression during the chronic phase of the disease (Marin-Neto et al., 2007; Nunes et al., 2013; Viotti et al., 2014). Overall, the prevailing evidence indicates that parasite persistence is fundamental for triggering and sustaining pathogenic processes.

## 2 The predicament in demonstrating the efficacy of antitrypanosomal therapy in chronic Chagas disease

One of the hurdles in Chagas disease is judging when a cure has been successful after antitrypanosomal therapy using the current criterion, which is the conversion of serologic response to negative (seroconversion) confirmed by conventional serologic tests. In acute Chagas disease, including congenitally transmitted infections, high cure rates have been reported (Meymandi et al., 2018) and serologic tests become negative relatively quickly after antitrypanosomal treatment. In a recent study of pediatric patients with Chagas disease treated with benznidazole, 29 out of 38 children aged ≤ 2 years seroconverted during a posttreatment follow-up observation period of 36 months (Moscatelli et al., 2019). This effectiveness of antitrypanosomal therapy is not detected in chronic Chagas disease, where parasitemia in the blood is intermittent. The parasite triggers a strong antibody response resulting in seropositivity and high titers of immunoglobulin G (IgG) anti-*T. cruzi* antibodies in the chronic phase of the disease. The antibodies persist even after complete eradication of the parasite, and the decline of conventional antibody titers may take a long time in chronic Chagas disease (Murphy et al., 2021). Indeed, it can take 10 years to 20 years following treatment before adult patients in the chronic phase of the disease become seronegative as assessed by conventional serologic methods (Meymandi et al., 2018; Perez-Molina and Molina, 2018).

The available conventional tests were primarily developed for the diagnosis of Chagas disease infections in, for example, blood donors. Currently, the measurement of response to antitrypanosomal therapy is also based on the demonstration of seroconversion as measured by conventional serologic tests. However, seropositivity persists in chronic Chagas disease for a long time after antitrypanosomal treatment and is related to the duration of *T. cruzi* infection (Meymandi et al., 2018). That makes it challenging to demonstrate the effectiveness of an antitrypanosomal therapy, especially in the chronic phase of the disease, which points to the dilemma researchers face with clinical trials on chronic Chagas disease. Currently, there is no sensitive, specific, easy-to-use marker established for early monitoring of the efficacy of antitrypanosomal treatment. This might be why, in several clinical studies initiated by academic investigators, polymerase chain reaction (PCR) has been used to measure the response to antitrypanosomal therapy in chronic Chagas disease. However, negative PCR results are not indicative of the absence of infection with *T. cruzi* but demonstrate only the absence of circulating *T. cruzi* deoxyribonucleic acid (DNA) in the blood at the moment the blood was drawn for testing. Negative PCR results are not conclusive evidence of parasite clearance.

To address the dilemma, several potential markers for early evaluation of the response to antitrypanosomal therapy have been suggested, including parasite biomarkers (e.g., F2/3, F29 antigens, and anti-Ag13), host response biomarkers (e.g., ApoA1 and endogenous thrombin potential), and immunological markers (e.g., INF-γ and IL12+ CD14+ cells). The inclusion of new technologies could also be helpful in identifying markers suitable as valid tools for use in clinical trials with new drugs (Ruiz-Lancheros et al., 2019).

### 2.1 Working with US FDA to define the efficacy endpoint for a clinical trial with nifurtimox in pediatric patients with Chagas disease

There is no guidance for industry on how to establish the effectiveness of drugs intended for the treatment of Chagas disease. In 2017, the United States Food and Drug Administration (US FDA) finalized recommendations on the use of serologic tests to reduce the risk of transmission of *T. cruzi* infection in blood and blood components for transfusion or for use in manufacturing a product^1^. Although this guidance document does not include recommendations for establishing the clinical effectiveness of drugs for the treatment of *T. cruzi* infections, it provides insights on blood donor testing for Chagas disease in the United States, including recommended serologic tests. Interestingly, the current cure criterion for Chagas disease is similar to the eligibility criterion for reentry of deferred blood donors as defined by the FDA in this guidance. This may be the basis for the FDA’s current request for results from two different tests for *T. cruzi* antibodies to determine the success of antitrypanosomal therapy in clinical trials in the pediatric population.

This position had to be taken into account in the planning of a confirmatory clinical trial in pediatric patients with Chagas disease to obtain marketing authorization for a new nifurtimox tablet formulation in the United States.

Consistent with the literature, the FDA considers positive PCR results as meaningful evidence of infection, but negative PCR results are not indicative of the absence of infection with *T. cruzi* and demonstrate only the absence of circulating *T. cruzi* DNA in the blood at the moment when the blood was drawn for testing (Alonso-Padilla et al., 2020; Sulleiro et al., 2020; Malone et al., 2021). For early detection of treatment failure, continuous PCR testing after antitrypanosomal therapy is suggested in patients with chronic Chagas disease (Sulleiro et al., 2020), but a PCR negative result at a single test is not definite proof of successful treatment.

The FDA considers qualitative serologic tests as adequate for determining a cure based on seroconversion from antibody-positive to antibody-negative status as a marker to predict parasite clearance. Seroreduction based on a reduction in antibody titer, by the indirect hemagglutination assay (IHA), is also an appropriate surrogate marker of efficacy.

Because in the confirmatory clinical trial with nifurtimox in pediatric patients with Chagas disease the period of the posttreatment follow-up was limited to 12 months, a surrogate endpoint of an at least 20% reduction in enzyme-linked immunosorbent assay (ELISA) optical density compared to baseline (seroreduction) was proposed as the primary measure of the effectiveness of nifurtimox; and this was discussed with the FDA before initiating the trial. To assess the response to nifurtimox treatment, we differentiated between seroconversion, the current standard criterion for cure, and seroreduction for patients who did not seroconvert within the study observation period, measured by two ELISA tests.

### 2.2 The surrogate endpoint of seroreduction

The persistence in *T. cruzi* seropositivity even after the successful treatment of chronic Chagas disease makes it extremely challenging, if not impossible, to interpret the outcome of clinical trials within a reasonable and feasible follow-up period. Therefore, seroconversion is considered not attainable within the time frame of clinical trials to confirm the efficacy of antitrypanosomal therapy, particularly in chronic Chagas disease (Ruiz-Lancheros et al., 2019; Malone et al., 2021), which places emphasis on the need for new biomarkers.

A continuous decrease in antibody titers precedes the complete reversion of the serologic response to negative, as measured by conventional assays, and from a clinical perspective indicates an evolution toward a cure (Moscatelli et al., 2019; Jurado Medina et al., 2021). Studies have shown that a reduction in antibody levels can be used to monitor the early impact of antitrypanosomal treatment (Sosa Estani et al., 1998; Fabbro et al., 2007; Viotti et al., 2011; Niborski et al., 2016; Sguassero et al., 2018; Moscatelli et al., 2019).

In a Phase III trial with 330 children with Chagas disease, we used seroreduction measured by two conventional ELISAs—recombinant ELISA (Chagatest ELISA recombinante v3.0; Wiener lab, Rosario, Argentina) and lysate ELISA (Chagatest ELISA lisado; Wiener lab, Rosario, Argentina)—as a measure of response to treatment with nifurtimox at the 12-month posttreatment follow-up in addition to the accepted criterion of seroconversion (Altcheh et al., 2021). This surrogate was considered acceptable by the FDA due to the fact that analysis of data from a study with benznidazole to treat children aged 6 years to 12 years with Chagas disease showed a 21% decrease in mean optical density for benznidazole versus no change for placebo at 12 months posttreatment (Sosa Estani et al., 1998), and that the seroreduction at 12 months correlated with seroconversion as measured by the same ELISA at the 4-year follow-up. Benznidazole was approved in the United States in 2017 using the Accelerated Approval Pathway for the treatment of pediatric patients aged 2 years to 12 years with Chagas disease; for full approval, a further study was requested to verify and describe the anticipated clinical benefits of benznidazole.

With this endpoint of seroreduction in addition to seroconversion, 32.9% of the patients (95% confidence interval [CI] 26.4% to 39.3%) showed a response to nifurtimox treatment administered for 60 days 12 months after the end of treatment in the Phase III trial (Altcheh et al., 2021). Response to nifurtimox treatment determined by conventional ELISA at the 12-month follow-up was consistent for recombinant ELISA and lysate ELISA. A dose–response relationship between the two nifurtimox treatment regimens was observed using the proportion of patients responding by obtaining either seroconversion or seroreduction measured in the two conventional ELISAs. The statistically significant difference observed for the conventional ELISAs comparing the two treatment regimens (32.9% vs. 18.9% in serologic response to nifurtimox treatment of the 60-day and the 30-day treatment regimens, respectively) tested using an asymptotic two-sided 95% CI for the difference between two independent proportions (Altcheh et al., 2021), precluded variability associated with random error for the recombinant and lysate ELISA tests. An increase in seroreduction was seen in other posttreatment follow-ups, and approximately 50% of the patients reached a serologic response to 60-day nifurtimox treatment by the 4-year follow-up (Altcheh et al., 2023). As reported by Moscatelli et al. (2019), there was a higher probability of seroreduction in younger patients. For the overall study population, the number of children who reached either seroreduction or seroconversion by the 4-year posttreatment with nifurtimox increased by approximately 30% from the 12-month follow-up (Altcheh et al., 2023). Interestingly, seroconversion measured by two types of assay could not be observed at the 4-year posttreatment follow-up in children aged ≥ 6 years at randomization, but the number of patients with seroreduction continuously increased (Altcheh et al., 2023). This may indicate that a 4-year posttreatment follow-up is not enough to observe seroconversion in this pediatric age group; this is supported by the median survival time of anti-*T. cruzi* antibodies of 90 months reported by Moscatelli et al. (2019) in children aged 3 years to 12 years.

In contrast to the serologic tests, the PCR results for the two treatment regimens did not differ significantly—93.6% and 91.9% of the patients treated for 60 days and 30 days with nifurtimox, respectively, were PCR negative 12 months posttreatment (Altcheh et al., 2021)—and, therefore, did not discriminate between the two nifurtimox regimens. The majority of the patients in both treatment groups showed continuous negative qPCR results during the additional 3-year follow-up (Altcheh et al., 2023).

Interestingly, only two (0.6%) of the 330 children included and diagnosed with Chagas disease based on ELISA serologic tests showed electrocardiogram abnormality (bradycardia) as a sign of Chagas disease at entry into the study, and bradycardia, respiratory arrhythmia, and a slight deviation of the QRS axis were reported in three children (1.9%) 12 months after the end of treatment with nifurtimox. All of these findings were considered non-pathological and not clinically significant by the investigator (Altcheh et al., 2021). No other clinical symptoms typical of Chagas disease were observed in any other study participant at baseline or at the 12-month posttreatment follow-up, and none of the study participants who were followed up for an additional 3 years reported any clinical signs and symptoms.

## 3 Discussion

Development of new, safe, and efficacious drugs for the treatment of Chagas disease is challenging due to several aspects that include but are not limited to the following:

1. Chagas disease is one of the neglected tropical diseases that are defined by the World Health Organization (WHO) as “a diverse group of 20 conditions that are mainly prevalent in tropical areas, where they mostly affect impoverished communities and disproportionately affect women and children. These diseases cause devastating health, social, and economic consequences to more than one billion people.”^2^ Such diseases are almost entirely absent from the global health agenda.
2. Currently, there are no markers for either the early assessment of response to antitrypanosomal therapy in the chronic phase of Chagas disease or for the prognosis of an infected individual.
3. The current standard criterion for the successful treatment of Chagas disease is seroconversion measured by conventional serologic tests. However, these tests were primarily developed for diagnostic purposes, and, consequently, such screening assays are optimized to pick up low-level antibodies indicative of past infections, and therefore cannot be instrumental for monitoring purposes (Zrein et al., 2018). Because anti-*T. cruzi* antibodies persist long-term, even after treatment, conventional serology remains reactive many years after treatment. Therefore, it can take years after antitrypanosomal treatment before patients with chronic Chagas disease are considered cured according to the current criterion. This makes measurement of treatment success using conventional serologic tests lengthy and challenging, particularly for drug development in Chagas disease.

These factors may have contributed to the fact that currently only two pharmaceuticals are approved for the treatment of Chagas disease: nifurtimox and benznidazole. Both drugs were launched in the 1970s and are recommended by the Pan American Health Organization (PAHO) and WHO in their *Guidelines for the diagnosis and treatment of Chagas disease*, issued in 2019^3^. Apart from strategies and methods for screening for Chagas disease and diagnosing patients, the guidelines provide recommendations for prescribing trypanocidal treatments in pediatric and adult patients depending on the phase of the disease. However, the guidelines do not detail recommendations on the laboratory tests to be used for regular and continuous posttreatment monitoring of the patients.

In children with chronic Chagas disease, seroconversion measured by conventional serologic assays is considered equivalent to a therapeutic response to antitrypanosomal therapy. However, there is a low degree of certainty on the validity of seroconversion and negativity in parasitemia as surrogates for clinical benefit, for example decrease in disease complications such as the development of heart disease or death. This further challenges designing clinical trials, particularly in adult patients with indeterminate chronic Chagas disease.

In the Phase III trial, we used a surrogate endpoint of seroreduction in addition to seroconversion measured by conventional ELISA tests to provide evidence of the effectiveness of nifurtimox for the treatment of children with Chagas disease. The two efficacy outcome measures allowed us to differentiate between the effectiveness of the two nifurtimox treatment regimens, whereas PCR did not. This efficacy surrogate endpoint used in the Phase III trial was accepted by the FDA in conjunction with other measures for efficacy, resulting in the approval of Lampit (nifurtimox) in the United States in 2020 under the provisions of Accelerated Approval Regulations (21 CFR 314.500). The data on seroconversion during the additional 3-year posttreatment follow-up confirmed the clinical benefit of nifurtimox in pediatric patients with Chagas disease and led to the full approval of Lampit (nifurtimox) in the United States in 2023 for use in pediatric patients (birth to <18 years of age and weighing at least 2.5 kg). for the treatment of Chagas disease (American trypanosomiasis) caused by *T. cruzi*.

Recent findings indicate that a novel antibody profiling multiplex assay, the MultiCruzi ELISA test, was able to reliably detect antibody signals in children diagnosed with Chagas disease who were treated with nifurtimox or benznidazole. The assay, along with mathematically driven approaches, could predict seronegative conversion earlier than a conventional serologic ELISA test (Jurado Medina et al., 2021). Based on our experience, using tests that are not approved by health authorities and not commercially available as the primary measure of efficacy in confirmatory clinical trials conducted for regulatory purposes could pose a challenge.

We suggest that working closely with health authorities and thoroughly discussing appropriate measures of efficacy is essential in the development of potential new therapies for Chagas disease.

## Data availability statement

The datasets presented in this study can be found in online repositories. The names of the repository/repositories and accession number(s) can be found below: https://clinicaltrials.gov/ct2/show/NCT02625974?term=nifurtimox&cond=Chagas+Disease&draw=2&rank=1.

## Ethics statement

The studies involving humans were approved by Hospital de Niños Ricardo Gutiérrez, Comité de Ética en Investigación del Hospital de Niños R. Gutiérrez, Gallo 1330, C1425EFD Buenos Aires, and 15 further ECs. The studies were conducted in accordance with the local legislation and institutional requirements. Written informed consent for participation in this study was provided by the participants’ legal guardians/next of kin.

## Author contributions

UG wrote the initial draft of the manuscript. M-LR critically reviewed and edited the manuscript. All authors contributed to the article and approved the submitted version.

## Footnotes

1 US Department of Health and Human Services, Food and Drug Administration, Center for Biologics Evaluation and Research. Use of Serological Tests to Reduce the Risk of Transmission of *Trypanosoma cruzi* Infection in Blood and Blood Components. Guidance for Industry. December 2017

2 World Health Organization. Health topics. Neglected tropical diseases (NTDs). Available at: https://www.who.int/health-topics/neglected-tropical-diseases#tab=tab_1. Accessed: March 10, 2023.

3 PAHO/WHO. Guidelines for the diagnosis and treatment of Chagas disease (2019). Available at: https://iris.paho.org/bitstream/handle/10665.2/49653/9789275120439_eng.pdf. Accessed: March 10, 2023.

## References

[B1] Alonso-Padilla J. Abril M. Alarcon de Noya B. Almeida I. C. Angheben A. Araujo Jorge T.. (2020). Target product profile for a test for the early assessment of treatment efficacy in Chagas disease patients: An expert consensus. PloS Negl. Trop. Dis. 14, e0008035. doi: 10.1371/journal.pntd.0008035 32324735 PMC7179829

[B2] Altcheh J. Castro L. Dib J. C. Grossmann U. Huang E. Moscatelli G.. (2021). Prospective, historically controlled study to evaluate the efficacy and safety of a new paediatric formulation of nifurtimox in children aged 0 to 17 years with Chagas disease one year after treatment (CHICO). PloS Negl. Trop. Dis. 15, e0008912. doi: 10.1371/journal.pntd.0008912 33412557 PMC7790535

[B3] Altcheh J. Sierra V. Ramirez T. Pinto Rocha J. J. Grossmann U. Huang E.. (2023). Efficacy and safety of nifurtimox in pediatric patients with Chagas disease: Results at 4-year follow-up in a prospective, historically controlled study (CHICO SECURE). Antimicrob. Agents Chemother. 67, e0119322. doi: 10.1128/aac.01193-22 36975790 PMC10112190

[B4] Alvarez-Hernández D. A. Franyuti-Kelly G. A. Díaz-López-Silva R. González-Chávez A. M. González-Hermosillo-Cornejo D. Vázquez-López R. (2018). Chagas disease: Current perspectives on a forgotten disease. Rev. Med. Hosp. Gen. (Mex.) 81, 154–164. doi: 10.1016/j.hgmx.2016.09.010

[B5] Balouz V. Aguero F. Buscaglia C. A. (2017). Chagas disease diagnostic applications: present knowledge and future steps. Adv. Parasitol. 97, 1–45. doi: 10.1016/bs.apar.2016.10.001 28325368 PMC5363286

[B6] Fabbro D. L. Streiger M. L. Arias E. D. Bizai M. L. del Barco M. Amicone N. A. (2007). Trypanocide treatment among adults with chronic Chagas disease living in Santa Fe city (Argentina), over a mean follow-up of 21 years: parasitological, serological and clinical evolution. Rev. Soc Bras. Med. Trop. 40, 1–10. doi: 10.1590/s0037-86822007000100001 17486245

[B7] Irish A. Whitman J. D. Clark E. H. Marcus R. Bern C. (2022). Update estimates and mapping for prevalence of Chagas disease among adults, United States. Emerg. Infect. Dis. 28, 1313–1320. doi: 10.3201/eid2807.212221 PMC9239882 35731040

[B8] Jurado Medina L. Chassaing E. Ballering G. Gonzalez N. Marque L. Liehl P.. (2021). Prediction of parasitological cure in children infected with *Trypanosoma cruzi* using a novel multiplex serological approach: an observational, retrospective cohort study. Lancet Infect. Dis. 21, 1141–1150. doi: 10.1016/S1473-3099(20)30729-5 33836157

[B9] Malone C. J. Nevis I. Fernandez E. Sanchez A. (2021). A rapid review on the efficacy and safety of pharmacological treatments for Chagas Disease. Trop. Med. Infect. Dis. 6, 128. doi: 10.3390/tropicalmed6030128 34287382 PMC8293415

[B10] Marin-Neto J. A. Cunha-Neto E. Maciel B. Simoes M. (2007). Pathogenesis of chronic Chagas heart disease. Circulation 115, 1109–1123. doi: 10.1161/circulationaha.106.624296 17339569

[B11] Meymandi S. Hernandez S. Park S. Sanchez D. R. Forsyth C. (2018). Treatment of Chagas disease in the United States. Curr. Treat. Options Infect. Dis. 10, 373–388. doi: 10.1007/s40506-018-0170-z 30220883 PMC6132494

[B12] Moscatelli G. Moroni S. Garcia Bournissen F. Gonzalez N. Ballering G. Schijman A.. (2019). Longitudinal follow up of serological response in children treated for Chagas disease. PloS Negl. Trop. Dis. 13, e0007668. doi: 10.1371/journal.pntd.0007668 31465522 PMC6715178

[B13] Murphy N. Cardinal M. V. Bhattacharyya T. Enriquez G. F. Macchiaverna N. P. Alvedro A.. (2021). Assessing antibody decline after chemotherapy of early chronic Chagas disease patients. Parasitol. Vectors. 14, 543. doi: 10.1186/s13071-021-05040-6 PMC8527601 34670602

[B14] Niborski L. L. Grippo V. Lafon S. O. Levitus G. Garcia-Bournissen F. Ramirez J. C.. (2016). Serological based monitoring of a cohort of patients with chronic Chagas disease treated with benznidazole in a highly endemic area of northern Argentina. Mem. Inst. Oswaldo Cruz. 111, 365–371. doi: 10.1590/0074-02760160006 27223650 PMC4909034

[B15] Nunes M. C. Dones W. Morillo C. A. Encina J. J. Ribeiro A. L. (2013). Chagas disease: an overview of clinical and epidemiological aspects. J. Am. Coll. Cardiol. 62, 767–776. doi: 10.1016/j.jacc.2013.05.046 23770163

[B16] Perez-Molina J. A. Molina I. (2018). Chagas disease. Lancet. 391, 82–94. doi: 10.1016/S0140-6736(17)31612-4 28673423

[B17] Ruiz-Lancheros E. Chatelain E. Ndao M. (2019). “Chagas disease treatment efficacy biomarkers: Myths and realities,” in Chagas disease - A clinical approach. Eds. Altcheh J. Freilij H. (Cham, Switzerland: Springer), 323–349.

[B18] Sguassero Y. Roberts K. N. Harvey G. B. Comande D. Ciapponi A. Cuesta C. B.. (2018). Course of serological tests in treated subjects with chronic *Trypanosoma cruzi* infection: A systematic review and meta-analysis of individual participant data. Int. J. Infect. Dis. 73, 93–101. doi: 10.1016/j.ijid.2018.05.019 29879524 PMC6069672

[B19] Sosa Estani S. Segura E. L. Ruiz A. M. Velazquez E. Porcel B. M. Yampotis C. (1998). Efficacy of chemotherapy with benznidazole in children in the indeterminate phase of Chagas' disease. Am. J. Trop. Med. Hyg. 59, 526–529. doi: 10.4269/ajtmh.1998.59.526 9790423

[B20] Sulleiro E. Silgado A. Serre-Delcor N. Salvador F. Tavares de Oliveira M. Moure Z.. (2020). Usefulness of real-time PCR during follow-up of patients treated with benznidazole for chronic Chagas disease: experience in two referral centers in Barcelona. PloS Negl. Trop. Dis. 14, e0008067. doi: 10.1371/journal.pntd.0008067 32069287 PMC7048293

[B21] Viotti R. Alarcon de Noya B. Araujo-Jorge T. Grijalva M. J. Guhl F. Lopez M. C.. (2014). Towards a paradigm shift in the treatment of chronic Chagas disease. Antimicrob. Agents Chemother. 58, 635–639. doi: 10.1128/AAC.01662-13 24247135 PMC3910900

[B22] Viotti R. Vigliano C. Alvarez M. G. Lococo B. Petti M. Bertocchi G.. (2011). Impact of aetiological treatment on conventional and multiplex serology in chronic Chagas disease. PloS Negl. Trop. Dis. 5, e1314. doi: 10.1371/journal.pntd.0001314 21909451 PMC3167788

[B23] Zrein M. Granjon E. Gueyffier L. Caillaudeau J. Liehl P. Pottel H.. (2018). A novel antibody surrogate biomarker to monitor parasite persistence in *Trypanosoma cruzi*-infected patients. PloS Negl. Trop. Dis. 12, e0006226. doi: 10.1371/journal.pntd.0006226 29425201 PMC5823467

